# Research on the Premotor Symptoms of Parkinson’s Disease: Clinical and Etiological Implications

**DOI:** 10.1289/ehp.1306967

**Published:** 2013-08-09

**Authors:** Honglei Chen, Edward A. Burton, G. Webster Ross, Xuemei Huang, Rodolfo Savica, Robert D. Abbott, Alberto Ascherio, John N. Caviness, Xiang Gao, Kimberly A. Gray, Jau-Shyong Hong, Freya Kamel, Danna Jennings, Annette Kirshner, Cindy Lawler, Rui Liu, Gary W. Miller, Robert Nussbaum, Shyamal D. Peddada, Amy Comstock Rick, Beate Ritz, Andrew D. Siderowf, Caroline M. Tanner, Alexander I. Tröster, Jing Zhang

**Affiliations:** 1 National Institute of Environmental Health Sciences, National Institutes of Health, Department of Health and Human Services, Research Triangle Park, North Carolina, USA; 2 Pittsburgh Institute for Neurodegenerative Diseases, University of Pittsburgh, Pittsburgh, Pennsylvania, USA; 3 Veterans Affairs Pacific Islands Health Care System, Honolulu, Hawaii, USA; 4 Pennsylvania State University–Milton S. Hershey Medical Center, Hershey, Pennsylvania, USA; 5 Mayo Clinic, Rochester, Minnesota, USA; 6 University of Virginia School of Medicine, Charlottesville, Virginia, USA; 7 Harvard School of Public Health, Boston, Massachusetts, USA; 8 Mayo Clinic Arizona, Scottsdale, Arizona, USA; 9 Institute for Neurodegenerative Disorders, New Haven, Connecticut, USA; 10 Emory University, Atlanta, Georgia, USA; 11 Institute for Human Genetics, University of California, San Francisco, San Francisco, California, USA; 12 Parkinson’s Action Network, Washington, DC, USA; 13 University of California, Los Angeles, Los Angeles, California, USA; 14 Avid Radiopharmaceuticals, Philadelphia, Pennsylvania, USA; 15 The Parkinson Institute, Sunnyvale, California, USA; 16 Department of Health Research and Policy, Stanford University School of Medicine, Stanford, California, USA; 17 Barrow Neurological Institute, Phoenix, Arizona, USA; 18 University of Washington, Seattle, Washington, USA

## Abstract

Background: The etiology and natural history of Parkinson’s disease (PD) are not well understood. Some non-motor symptoms such as hyposmia, rapid eye movement sleep behavior disorder, and constipation may develop during the prodromal stage of PD and precede PD diagnosis by years.

Objectives: We examined the promise and pitfalls of research on premotor symptoms of PD and developed priorities and strategies to understand their clinical and etiological implications.

Methods: This review was based on a workshop, Parkinson’s Disease Premotor Symptom Symposium, held 7–8 June 2012 at the National Institute of Environmental Health Sciences in Research Triangle Park, North Carolina.

Discussion: Research on premotor symptoms of PD may offer an excellent opportunity to characterize high-risk populations and to better understand PD etiology. Such research may lead to evaluation of novel etiological hypotheses such as the possibility that environmental toxicants or viruses may initiate PD pathogenesis in the gastrointestinal tract or olfactory bulb. At present, our understanding of premotor symptoms of PD is in its infancy and faces many obstacles. These symptoms are often not specific to PD and have low positive predictive value for early PD diagnosis. Further, the pathological bases and biological mechanisms of these premotor symptoms and their relevance to PD pathogenesis are poorly understood.

Conclusion: This is an emerging research area with important data gaps to be filled. Future research is needed to understand the prevalence of multiple premotor symptoms and their etiological relevance to PD. Animal experiments and mechanistic studies will further understanding of the biology of these premotor symptoms and test novel etiological hypothesis.

Citation: Chen H, Burton EA, Ross GW, Huang X, Savica R, Abbott RD, Ascherio A, Caviness JN, Gao X, Gray KA, Hong JS, Kamel F, Jennings D, Kirshner A, Lawler C, Liu R, Miller GW, Nussbaum R, Peddada SD, Comstock Rick A, Ritz B, Siderowf AD, Tanner CM, Tröster AI, Zhang J. 2013. Research on the premotor symptoms of Parkinson’s Disease: clinical and etiological implications. Environ Health Perspect 121:1245–1252; http://dx.doi.org/10.1289/ehp.1306967

## Introduction

Parkinson’s disease (PD) is the second most prevalent neurodegenerative disease and severely affects quality of life. More than 1 million older U.S. adults live with PD, and the number will double by the year 2030 (Bach et al. 2011). Clinical diagnosis of PD is currently based on the presence of motor dysfunction including rest tremor, bradykinesia, and rigidity. PD patients also suffer from a wide range of non-motor symptoms—including hyposmia (poor sense of smell), gastrointestinal dysfunction, psychiatric features (e.g., depression, anxiety, psychosis), sleep disorders, and mild-to-severe cognitive impairment—many of which are disabling and can be difficult to treat (Coelho and Ferreira 2012; Fernandez 2012) and greatly jeopardize the quality of life of PD patients (Storch et al. 2013). Pathologically, PD has been characterized by the loss of dopamine neurons in the substantia nigra pars compacta, which underlies motor dysfunction, and by the presence of Lewy bodies in selected regions of the brain.

The cardinal motor signs of PD become clinically evident when approximately 50% of the dopaminergic neurons in the substantia nigra are lost (Fearnley and Lees 1991). Despite symptomatic therapies for dopamine deficiency–related motor features, the disease continues to progress and often leads to severe mental and physical disabilities (Coelho and Ferreira 2012; Shulman et al. 2008) and increased mortality (Chen et al. 2006; Willis et al. 2012). To date, none of the available treatments can halt or reverse the pathological and clinical progression of PD, and novel strategies are needed. Research on disease-modifying strategies would be greatly assisted by the identification of high-risk populations.

Recent interest has focused on the non-motor symptoms of PD, some of which may predate motor signs and clinical diagnosis by years (“premotor symptoms” of PD). Accumulating epidemiological and clinical evidence suggests that hyposmia (Ross et al. 2008), constipation (Abbott et al. 2007; Gao X et al. 2011; Savica et al. 2009), depression (Bower et al. 2010; Fang et al. 2010; Ishihara-Paul et al. 2008; Shiba et al. 2000), anxiety (Bower et al. 2010; Ishihara-Paul et al. 2008; Shiba et al. 2000; Weisskopf et al. 2003), rapid eye movement sleep behavior disorder (RBD) (Claassen et al. 2010; Iranzo et al. 2006; Postuma et al. 2009; Schenck et al. 1996), excessive daytime sleepiness (EDS) (Abbott et al. 2005; Gao J et al. 2011), and autonomic dysfunction (Goldstein 2010) may occur well before the appearance of the classic motor dysfunction of PD. Evidence comes primarily from large prospective population-based cohort studies that were initially established for research on cancer and cardiovascular disease, such as the Honolulu Asia Aging Study (HAAS) (Ross et al. 2008) and the Health Professionals Follow-up Study (Gao X et al. 2011), and from retrospective examinations of archived medical records of PD cases and controls such as the Rochester Epidemiology Project (Savica et al. 2009). These findings are summarized in Table 1. A recent meta-analysis also confirmed that constipation and mood disorders were associated with higher risk of PD (Noyce et al. 2012). Hyposmia, RBD, and EDS were not included in this meta-analysis because risk estimates either were not available or were available from only one study.

**Table 1:** Prospective evidence on selected premotor symptoms in large population-based studies.

Symptom	Study/reference	Age [years (mean ± SD and/or range)]	Years of follow-up	No. of cases	Assessment	Primary results [RR/HR/OR (95% CI)]	Timeline [years prior to PD]^*a*^
Hyposmia	HAAS, men only (Ross et al. 2008)	79.7 ± 4.1 (71–95)	≤ 8 years	35	BSIT score < 6	Lowest vs. top two quartiles: 5.2 (1.5–25.6) for the first 4 years, 0.3 (0.0–2.7) for the second 4 years of follow-up	Within 4 years
Constipation^*b*^	HAAS, men only (Abbott et al. 2007)	60 (51–75)	≤ 24 years	96	Self-reported bowel movement frequency	< 1/day vs. > 2/day: 4.5 (1.2–16.9)	Could be ≥ 12 years
	REP (Savica et al. 2009)	––	––	196	Medical record review: constipation diagnosis or laxative use	2.5 (1.5–4.1)	Could be ≥ 20 years
	HPFS, men only (Gao X et al. 2011)	~ 54–89	≤ 6 years only	156	Self-reported bowel movement frequency	≤ 2/week vs. daily: 5.0 (2.6–9.6)	6 years and probably more
	NHS, women only (Gao X et al. 2011)	~ 36–61	≤ 24 years	402	Self-reported bowel movement frequency	Within 6 years of follow up: 2.2 (0.8–6.1); no association beyond 6 years	May limit to 6 years of follow-up
Daytime sleepiness^*c*^	HAAS men only (Abbott et al. 2005)	77 (71–93)	≤ 8 years	43	Self-report: single question	2.8 (1.1–6.4)	0.5–4.9 years
	NIH-AARP DH (Gao J et al. 2011)	52–71	4–10 years	770	Self-reported hours of daytime napping	≥ 1 vs. 0 hr: 1.5 (1.2–1.9)	4–10 years
RBD^*d*^	Mayo Clinic (Claassen et al. 2010)	21–60	Only if ≥ 15 years	9 PD of 27 RBD	Clinical diagnosis	—	15–50 years
	Barcelona, Spain (Iranzo et al. 2006)	74.1(61–86)	> 2 years	7 PD of 44 RBD	Clinical diagnosis	—	Could be 6–18 years
	Minnesota, men only (Schenck et al. 1996)	54.5	—	11 PD of 29 RBD	Clinical diagnosis	—	Could be by 10–29 years
	Montreal (Postuma et al. 2009)	65.4 ± 9.3	—	19 PD of 93 RBD	Clinical diagnosis	—	On average preceded by 11 years
Depression	EPIC-Norfolk (Ishihara-Paul et al. 2008)	41–80	Median, 8 years	175	Structured questionnaire	Lifetime major depression 2.1 (1.4–2.9)	Similar results for first episode of depression before or after 40 years of age
	REP (Bower et al. 2010)	48.3 (20–69)	Mean, 29 years (up to 45 years)	156	MMPI	Quartile 4 vs. quartiles 1–3: 1.16 (0.81–1.66)
	REP (Shiba et al. 2000)		51 years (8–87 years)	196	Medical record review	1.9 (1.1–3.2)	Within 5 years
Anxiety	EPIC-Norfolk (Ishihara-Paul et al. 2008)	41–80	Median, 8 years	175	Structured questionnaire	2.7 (1.5–4.7)
	HPFS, men only (Weisskopf et al. 2003)	56.0 (42–77)	≤ 12 years	189	Crown–Crisp anxiety index	Score ≥ 4 vs. 0–1: 1.5 (1.0–2.1)	Could be > 2 years
	REP (Bower et al. 2010)	48.3 (20–69)	Mean, 29 years (up to 45 years)	156	MMPI	Quartile 4 vs. quartiles 1–3: 1.63 (1.16–2.27)/ men 2.03 (1.28–3.24)/ women 1.29 (0.79–2.10)
	REP (Shiba et al. 2000)		51 years (8–87)	196	Medical record review	2.2 (1.4–3.4); slightly attenuated, even restricted to > 20 years before index date	Could be > 20 years
Abbreviations: BSIT, brief smell identification test; EPIC, European Prospective Investigation into Cancer; HAAS, Honolulu Asia Aging Study; HPFS, Health Professionals Follow-up Study; HR, Hazard Ratio; MMPI, Minnesota Multiphasic Personality Inventory; NHS, Nurse’s Health Study; NIH-AARP DH, National Institutes of Health-AARP Diet and Health study; OR, odds ratio; RBD, rapid eye movement sleep behavior disorder; REP, Rochester Epidemiology Project; RR, relative risk. ^***a***^The time from measurement of non-motor symptoms to PD diagnosis are best estimates; these estimates could, however, be misleading because they were bounded by the length of follow-up and inclusion criteria. ^***b***^The HAAS and HPFS/NHS used the frequency of bowel movement as an indicator for constipation. ^***c***^The NIH-AARP DH used daytime napping duration as a surrogate for daytime sleepiness. ^***d***^Based on follow-ups of RBD patients.							

The hypothesis that premotor symptoms precede the motor signs of PD is broadly compatible with neuropathological findings reported by Braak et al. (2003). This work, although controversial (Burke et al. 2008), suggests that deposition of α-synuclein in the form of Lewy bodies and Lewy neurites develops in the PD brain in six sequential stages. α-Synuclein pathology begins in the dorsal motor nucleus of the vagus and glossopharyngeal nerves and the anterior olfactory nucleus in stage 1, extends to the locus ceruleus and caudal raphe nuclei in the pons (stage 2), then to the substantia nigra (stage 3), to the temporal mesocortex (stage 4), and finally to the neocortex (stages 5–6). A later extension of this hypothesis further posits that the synucleinopathy may even first develop in the enteric nerves in the gut and later spread along the vagus nerve into the brain (Hawkes et al. 2007, 2009). Importantly, according to the Braak hypothesis, the irreversible loss of dopamine neurons in the substantia nigra and associated progressive motor dysfunction may not be evident until Braak stages 3 and 4. Although the Braak hypothesis is not universally supported (Burke et al. 2008; Dickson et al. 2010), it presents the intriguing possibility that the extra-nigra, nondopaminergic pathologies are intrinsic to early PD pathogenesis and that premotor symptoms could well be part of the disease’s natural history (Hawkes et al. 2010).

Growing evidence on the importance of premotor symptoms, coupled with the Braak hypothesis, has generated substantial interest in understanding the origins and consequences of these symptoms. Clinical research primarily has focused on evaluating premotor symptoms and other factors as markers for the future development of PD, a subject elegantly reviewed by Berg et al. (2012). Another potential line of inquiry is based on the idea that the presence of multiple premotor symptoms in the same individual represents common underlying pathogeneses that may eventually lead to PD, and thus premotor symptoms may provide a unique opportunity to understand the etiology of PD (Hawkes et al. 2007, 2009). Despite this potential promise, little research has been carried out to understand the etiological implications of the premotor symptoms of PD.

This review was based on a workshop, Parkinson’s Disease Premotor Symptom Symposium, held 7–8 June 2012 at the National Institute of Environmental Health Sciences in Research Triangle Park, North Carolina.

A comprehensive review of the clinical and epidemiological evidence for the existence of these premotor symptoms in PD is outside the scope of this review. Instead, we focus on outlining the promises and pitfalls of the concept of premotor symptoms and on developing research priorities and strategies for understanding the clinical and etiological implications of these symptoms. Although these symptoms can also develop after the clinical diagnosis of PD, for this review, we focus on the period prior to the emergence of diagnostic motor abnormalities.

## Identification of High-Risk Populations

Several important studies have tested the hypothesis that premotor symptoms, coupled with neuroimaging, may lead to early identification, or even diagnosis, of PD (Lang 2011; Tolosa et al. 2009). Preliminary results have been published from the Prospective Evaluation of Risk Factors for Idiopathic Parkinson’s Syndrome (PRIPS) study (Berg et al. 2013) and the Parkinson At-Risk Syndrome (PARS) study (Siderowf et al. 2012). PRIPS aimed to test a two-stage screening strategy for early identification of PD cases utilizing the following predictors: hyposmia, PD family history, subtle motor impairment, and substantia nigra hyperechogenicity (SN+). This general population–​based cohort recruited 1,352 participants ≥ 50 years of age (mean age, 59 years). Hyposmia was evaluated using the Sniffin’ Stick test (Hummel et al. 2001) and SN+ by transcranial sonography. A total of 10 participants developed PD during approximately 3 years of follow-up. Hyposmia was strongly associated with the risk of developing PD with sensitivity and specificity both > 70%. The positive predictive value (PPV), however, was only 2%, in part due to the low PD incidence in this relatively young population. Combining hyposmia with other characteristics (e.g., family history or SN+) only slightly increased the PPV, but substantially decreased the sensitivity. Unlike the PRIPS study, PARS was conducted among a risk-enriched population in which 45% of the 4,999 study participants (mean age, 64 years) had a family history of PD. The study was designed to evaluate a two-stage strategy of at-risk identification: olfactory testing using the University of Pennsylvania Smell Identification Test (Doty et al. 1984), followed by dopamine-transporter (DAT) imaging. Although findings on the DAT scan portion are yet to be published, preliminary analyses showed that participants with hyposmia were more likely to have other non-motor features and to report changes in motor function (Siderowf et al. 2012).

Of the premotor symptoms, the PRIPS and PARS studies focused on hyposmia. The results from these studies, albeit preliminary, clearly show that an individual premotor symptom by itself is inadequate for early disease identification. This is actually what one would expect for a relatively rare disease such as PD because the PPV depends on the prevalence of prediagnostic cases in the target population.

The utility of combinations of premotor symptoms for early disease identification has been little explored and merits consideration. Although the underlying etiologies of premotor symptoms in the general population are likely diverse, the presence of multiple symptoms in individuals who later develop PD may reflect common or similar underlying pathologies, for example Lewy pathology in various sites of the brain, spinal cord, and autonomic nervous system. In support of this notion, in the HAAS, both the sense of smell (Ross et al. 2006) and bowel movement frequency (Abbott et al. 2007) were strongly related to incidental Lewy body disease among individuals without PD. Further, α-synuclein was identified from colon tissues of PD patients collected 2–5 years before PD motor onset (Shannon et al. 2012), but not in any of the controls.

**Figure 1:**
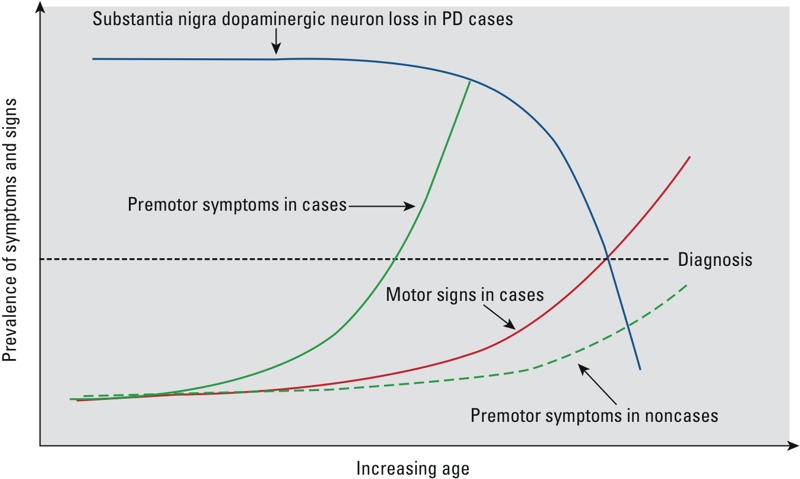
A hypothesis on the development of premotor symptoms among persons who will or will not develop PD in lifetime. The green lines represent the joint prevalence of multiple premotor symptoms by age; solid, future PD cases, and dashed for noncases. The red line represents motor signs among future PD patients. The blue line represents the loss of dopaminergic neurons in the substantia nigra pars compacta of PD patients which underlies cardinal motor signs. PD diagnosis is made based on cardinal motor signs (red) when approximately 50% of the dopaminergic neurons in the substantia nigra have been lost (threshold shown as the black dotted line). Individuals at high risk for PD (solid green line) will develop multiple premotor symptoms years before onset of PD motor signs; for individuals who will not develop PD (dashed green line), the joint prevalence of these symptoms remain at low level even at older age.

One may further hypothesize that among individuals who will develop PD, multiple premotor symptoms develop over time as a result of common pathologies and eventually become a clinically recognizable syndrome several years before PD diagnosis (Figure 1). In contrast, among individuals who will not develop PD, these symptoms may also exist, but they are more independent of each other and more randomly distributed over the entire life period. Therefore, the joint prevalence of multiple premotor symptoms in a low-risk population will be low. These hypotheses are yet to be systematically examined, but there are preliminary supportive data. Based on hyposmia, infrequent bowel movement, slow reaction time, and excessive daytime sleepiness, the HAAS showed that 2 of the 24 individuals with more than three of these symptoms developed PD within 4.6 years of follow-up, as compared with 8 of 852 for those with only one symptom (Berg et al. 2012). Preliminary evidence also comes from research in high-risk populations. Non-Parkinsonian family members of patients with the leucine-rich repeat kinase 2 (*LRRK2* G2019S) mutation showed more constipation and poorer color discrimination than controls (Marras et al. 2011). Therefore, preliminary evidence does suggest that multiple non-motor symptoms tend to cooccur among individuals at higher risk for PD.

It is also important to understand when multiple premotor symptoms become detectable in the prodromal stage of PD. Ideally, this should be investigated in large prospective cohorts with long follow-up and repeated measurements of multiple premotor symptoms. In reality, we have just begun to understand the temporal relationships between individual symptoms and PD by examining existing clinical data or data from prospective cohorts (Table 1). For example, several clinical studies have consistently documented RBD onset about 10–20 years before PD onset (Boeve and Saper 2006; Claassen et al. 2010; Iranzo et al. 2006; Postuma et al. 2009; Schenck et al. 1996). These were studies of RBD patients who were diagnosed by polysomnography, and it has yet to be determined whether this clinical observation can be generalized to the general elderly population where only questionnaire-based screening for probable RBD is possible (Postuma et al. 2012a). Data on the timing of other key premotor symptoms are limited or inconsistent. For example, several studies showed that constipation might precede PD clinical diagnosis by 10–20 years in men (Abbott et al. 2001; Gao X et al. 2011; Savica et al. 2009), but data are not consistent in women (Gao X et al. 2011; Savica et al. 2009). The population-based HAAS showed that hyposmia was highly predictive of PD onset within 4 years after symptom assessment (Ross et al. 2008). Two other studies among high-risk individuals (Ponsen et al. 2009; Postuma et al. 2011) showed that hyposmia predicted PD risk throughout the entire follow-up period of 5 years. Because the assessment of temporal relationship will be bounded by the length of follow-up, future studies should have longer periods of follow-up and repeated symptom assessments. More importantly, future studies should also investigate the temporal pattern of multiple premotor symptoms in prodromal PD cases.

Measuring premotor symptoms for neurodegeneration research represents a substantial challenge. Studies to date have mostly used simple methods to identify premotor symptoms, including methods such as self-reported symptoms, self-reported diagnoses, screening tests, and structured questionnaires. For example, the sense of smell is often measured with simple screening tests such as the Brief Smell Identification Test (Ross et al. 2008) or the Sniffin’ Stick Test (Hummel et al. 2001), and hyposmia is defined as a score below population norms. These simple methods have served well to establish the associations between premotor symptoms and PD. However, because most premotor symptoms are common in the elderly and are etiologically heterogeneous (Doty 2009; Leung et al. 2011), novel approaches are needed to assess various modalities of these symptoms and to identify patterns that are more specific to PD. Compared to other premotor symptoms, RBD is more specific; however, its diagnosis requires polysomnographic confirmation at sleep clinics. Several screening questionnaires for probable RBD (Boeve et al. 2011; Li et al. 2009; Postuma et al. 2012a; Stiasny-Kolster et al. 2007) have been developed and validated in clinical settings, but their validities in identifying RBD patients from the general population are yet to be evaluated.

A large prospective study reported subjective complaints of motor dysfunctions such as stiffness and tremor prior to PD diagnosis (de Lau et al. 2006). In fact, subtle motor abnormalities have been quantitatively documented among individuals at high risk for PD. For example, Mirelman et al. (2011) reported subtle gait changes among asymptomatic carriers of *LRRK2* mutation with quantified gait analyses under challenged conditions. Among RBD patients, Postuma et al. (2012b) documented multiple motor abnormalities on average 6–8 years prior to PD diagnosis, including voice and face akinesia, rigidity, abnormal gait, limb bradykinesia. Evaluation of subtle motor changes in addition to premotor symptoms may prove important in differentiating PD from other causes of non-motor symptoms. Further, the development and use of standardized assessment tools for non-motor and motor symptoms such as the National Institues of Health/National Institute of Neurological Disorders and Stroke (NIH/NINDS) common data elements for PD will greatly facilitate such research (NINDS 2013).

## Implications for Parkinson’s Etiology and Experimental Research

**Figure 2:**
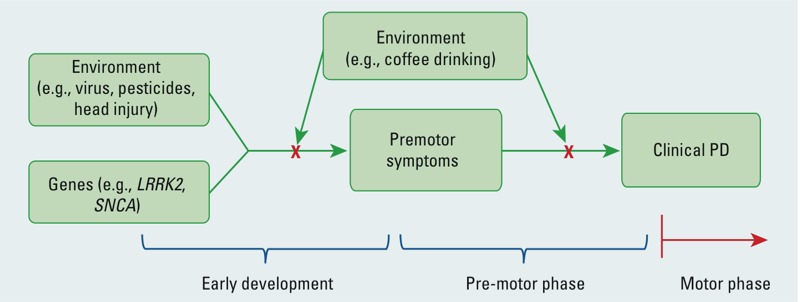
A hypothesis on risk factors, premotor symptoms, and PD. Environmental or genetic factors may initiate neurodegeneration through mechanisms such as neuroinflammation; in susceptible individuals, this may first lead to premotor symptoms years before PD clinical onset; if this neurodegeneration continues without effective intervention, premotor symptoms may eventually progress into overt PD; however, with interventions such as coffee drinking, this premotor progression may be halted before it becomes irreversible.

An inherent implication of research on premotor symptoms is that it may eventually lead to a better understanding of PD etiology (Hawkes et al. 2007, 2009). The concept that premotor symptoms represent intermediate phenotypes prior to overt PD may offer us a vehicle to understand the roles of genetics and environment in the early stages of PD development. For example, neurotoxicants or viruses may enter the body via the nasal cavity or the digestive tract (Hawkes et al. 2007, 2009), and, in susceptible individuals, may initiate Lewy pathology in the olfactory bulb or the enteric nerves (Doty 2008; Hawkes et al. 2007, 2009; Reichmann 2011); over time, this may lead to premotor symptoms such as hyposmia or constipation and may eventually progress to PD. It is therefore important to identify environmental and genetic factors associated with the presence of multiple premotor symptoms and, more importantly, to identify factors that may prevent the progression of premotor symptoms to clinical PD. This concept is illustrated in Figure 2.

To the best of our knowledge, no epidemiological study has examined common etiological factors for the presence of multiple non-motor symptoms. Preliminary data are available only on risk factors for individual symptoms. Postuma et al. (2012c) recently published the first report on environmental risk factors for RBD. In this multicenter study of 347 cases and 347 controls, RBD was positively associated with pesticide exposure and head injury. However, unlike PD, RBD was more common among smokers and was not related to caffeine intake. More studies have examined risk factors associated with hyposmia. All studies found that the risk of hyposmia increases with age and is higher in men than in women (Brämerson et al. 2004; Schubert et al. 2011, 2012; Siderowf et al. 2007, 2012; Vennemann et al. 2008). Data on smoking or coffee drinking and hyposmia are, however, preliminary and inconsistent (Brämerson et al. 2004; Schubert et al. 2011, 2012; Siderowf et al. 2007, 2012; Vennemann et al. 2008), although current smoking is associated with a higher risk of hyposmia in some studies (Schubert et al. 2012; Vennemann et al. 2008). In adults, the prevalence of constipation is higher in women and increases modestly with age (McCrea et al. 2009; Suares and Ford 2011). Other suspected risk factors for constipation include inadequate fluid or dietary fiber intake, less physical activity, concurrent use of certain medications, levels of thyroid hormone and progesterone, and a wide range of medical conditions including neurodegenerative diseases (Leung 2007; Leung et al. 2011). Therefore, risk factors for individual non-motor symptoms are diverse, and PD-related pathology is probably only a small contributor to the prevalence of each of these symptoms. Combining multiple premotor symptoms may rule out some diverse pathologies unrelated to PD. Further, PD is likely to be phenotypically and etiologically heterogeneous (van Rooden et al. 2009, 2011); and careful phenotyping of various PD motor and non-motor symptoms may help us understand the interrelationship among risk factors, premotor symptoms, and neurodegeneration.

Although human observational studies are essential to define premotor symptoms and their relationship to PD development, experimental studies are needed to understand the underlying biology and to examine novel etiological hypotheses. For example, could the gastrointestinal tract or the olfactory bulb be the sites of initial exposure to a pathogenic environmental agent (Reichmann 2011)? Does pathology progress anatomically, as the Braak hypothesis predicts (Pan-Montojo et al. 2012)? Does the selectivity and order of neurodegeneration in PD reflect differential sensitivity to environmental agents or some other mechanism of progression such as prion-like spreading (Dunning et al. 2012)? Experimental research that models prodromal PD may help answer these questions.

Animal models based on toxicants [e.g., 1-methyl-4-phenyl-1,2,3,6-tetrahydropyridine (MPTP), 6-hydroxydopamine (6-OHDA), paraquat, rotenone] and those based on genes [e.g., *SNCA* (α-synuclein), *LRRK2*, *PINK1* (PTEN-induced putative kinase 1), *PARK2* (parkin)] have been used for PD research (Bezard et al. 2012; Blesa et al. 2012). These models were developed to mimic features of late-stage PD such as dopaminergic neuron loss and motor dysfunction, or to recapitulate particular pathogenic processes such as neuroinflammation. The extent to which current models replicate the non-motor features of PD is incompletely known at present, although recent work has revealed some intriguing results suggesting abnormalities analogous to non-motor features of PD (Jellinger 2011; McDowell and Chesselet 2012; Smith et al. 2012).

Central to this work is the availability of validated methods to evaluate olfaction, gastrointestinal function, sleep disturbances, or depression/anxiety in experimental animals. The technical difficulties of reliably determining the presence or absence of these non-motor features in experimental animals are not trivial. Furthermore, the mechanistic relationship between abnormalities observed in the commonly used assays in experimental animals and the analogous symptoms in human patients is uncertain, especially for complex behavioral traits such as depression and anxiety. Nonetheless, ways to evaluate these non-motor symptoms have been reported in mice, rats, primates, and zebrafish. Each of these animals shows phylogenetic conservation of neuroanatomical structures involved in early Braak stages of PD pathology, suggesting that they might be employed as models to study premotor PD.

So far, a number of animal models of PD have shown either non-motor functional abnormalities or pathology outside the substantia nigra. Olfactory function has been shown to be abnormal in MPTP-treated rodents (Schintu et al. 2009), transgenic mice expressing α-synuclein under a neuronal regulatory element derived from the Thy1 gene (Thy1-αSyn) (Fleming et al. 2008), and mice expressing reduced levels of the vesicular monoamine transporter (VMAT) (Taylor et al. 2009). Sleep and circadian rhythm are known to be disrupted in MPTP-treated rodents (Laloux et al. 2008), rotenone-treated rats (García-García et al. 2005), and Thy1-αSyn mice and VMAT2-deficient mice (Taylor et al. 2009). Gastrointestinal function has been shown to be abnormal in MPTP-treated mice (Anderson et al. 2007), rotenone-treated rats (Drolet et al. 2009), Thy1-αSyn mice (Wang et al. 2008, 2012), SNCA PAC mice (which expresss mutant human α-synuclein from a P1 artificial chromosome containing its endogenous regulatory elements) (Kuo et al. 2010), and VMAT2-deficient mice (Taylor et al. 2009). These findings are of interest because they demonstrate that toxicant exposures and genetic manipulations used to induce motor signs of PD can also induce non-motor features. This suggests that at least some of the neuronal populations underlying non-motor symptoms share susceptibility with dopamine neurons to agents implicated in motor PD pathogenesis. This is consistent with a model in which common etiological mechanisms could underlie both motor and non-motor components of the disease.

Interestingly, a few of the models have shown ordered progression from non-motor to motor symptoms. Thy1-αSyn transgenic mice showed α-synuclein inclusions in the olfactory bulb and deficits in olfactory function on multiple tests by 3 months of age (Fleming et al. 2008). By this time point, animals also showed progressively worsening sleep abnormalities (Kudo et al. 2011) and progressive reduction in stool frequency (Wang et al. 2012). These changes preceded loss of striatal dopamine, which did not occur until 14 months of age (Lam et al. 2011). Similarly, VMAT2-deficient mice demonstrated progressive non-motor symptoms prior to the onset of motor deficits (Taylor et al. 2009). Gastrointestinal dysfunction was seen at 2 months of age, olfactory defects by 5 months, and anxiety-like behavior at 6 months. l-dihydroxyphenylalanine (l-DOPA)–responsive hypokinesia and loss of striatal tyrosine hydroxylase terminals were present by 18 months of age, and loss of nigral dopamine neurons worsened between 18 and 24 months (Caudle et al. 2007). Data from both models imply that a systemic abnormality affecting all cells can result in specific abnormalities of neuronal populations implicated in non-motor and motor PD with replication of some of the temporal course.

These data do not yet allow us to distinguish between a model for pathogenic progression in which the temporal course of the disease is dictated by the differential vulnerability of various neuronal groups to a systemic abnormality and an alternative model in which pathology spreads anatomically from one site of the nervous system to another to produce progressive symptoms. Much recent attention has been given to the idea that α-synuclein has prion-like properties and that α-synucleinopathy can spread from a site of initial pathology to other regions of the central nervous system (CNS) by axonal transport and cell-to-cell spread (Luk et al. 2012). In this regard, it is noteworthy that the pathology in both Thy1-αSyn mice (Fleming et al. 2004) and VMAT2-deficient mice (Taylor et al. 2009) is dependent on the presence of α-synuclein. However, several alternative explanations for the progression of disease are equally consistent with the available data and further studies will be necessary to determine whether progression can be arrested by interventions that prevent the transport or transmission of pathological α-synuclein species, or whether additional cellular factors dictate the differential vulnerability of neuronal groups involved in non-motor symptoms.

The hypothesis that an environmental agent could provoke pathology at an anatomical site of entry that then progresses to involve other structures, culminating in degeneration of the substantia nigra, has received some preliminary experimental support. For example, Jang et al. (2009) reported that, in mice, intranasally injected H5N1 influenza virus travelled from the enteric nervous system (ENS) into the CNS and eventually caused degeneration of dopaminergic neurons. Further, this sequence was accompanied by chronic neuroinflammation with microglial activation and elevated expression of cytokines and other proinflammatory biomarkers (Jang et al. 2012). These findings imply that initiating pathogenic events can provoke distinct secondary mechanisms underlying disease progression, with the important implication that environmental agents that trigger early events in PD pathogenesis may no longer be present at the end stage of the disease, when tissue samples are generally available for analysis.

The gastrointestinal tract is potentially an important site for exposure to environmental agents, and the suggestion that α-synuclein pathology in the ENS may be one of the first abnormalities in PD patients has promoted interest in the possibility of modeling pathology in the ENS and its subsequent progression to the CNS. Transgenic mice expressing human α-synuclein under its own regulatory elements showed prominent ENS pathology, but no progression to other features of PD (Kuo et al. 2010), suggesting that a second event was necessary to promote disease progression. Recent reports showed that intragastric rotenone caused α-synuclein aggregation in mice, following a staged pattern that was consistent with the Braak hypothesis (Pan-Montojo and Funk 2010; Pan-Montojo et al. 2010), and resection of the autonomic nerves prevented this progression (Pan-Montojo et al. 2012). These interesting observations are yet to be replicated by other laboratories, and their interpretation consequently remains speculative. However, the local microenvironment of the gastrointestinal tract remains a potentially significant factor in dictating initiating pathogenic events, and is worthy of further investigation. This might also encompass evaluation of the role of the gut microbiome, which could be experimentally manipulated in animal models to determine whether alterations can initiate PD pathology or modulate the time course of onset of pathology and progression. Although few empirical data exist regarding the role of the microbiome in PD, the microbiome influences the immune system, gastrointestinal mobility, and the metabolism of nutrients and other exogenous chemicals (Grenham et al. 2011), all of which may potentially contribute to the development of PD. Similarly, experimental animals could be exposed to toxicants through the gastrointestinal tract to evaluate whether etiologically implicated exogenous agents can provoke the earliest pathological changes of PD or modulate their appearance in experimental models. These studies could provide valuable mechanistic insights and generate further hypotheses to be addressed in human studies.

Finally, although this review focuses on PD, research on premotor symptoms may have broader implications because many of these symptoms have been linked to other neurological diseases. For example, hyposmia is associated with higher risk of cognitive decline and Alzheimer’s disease (Wilson et al. 2007, 2009), and RBD precedes Lewy body dementia and multiple system atrophy (Schenck et al. 2013). Further, olfactory dysfunction has been documented in schizophrenic patients and individuals at high risk for schizophrenia (Moberg et al. 2013). Therefore research on premotor symptoms may eventually provide novel insights into the natural history and etiology of neurodegeneration and related conditions in addition to PD, and into the complex interrelationships among these conditions.

## Conclusion

Premotor symptoms of PD may offer us an excellent opportunity to identify populations at higher risk for PD and to understand early disease etiology. Further research is needed to understand whether the presence of multiple premotor symptoms is predictive of PD. Animal experiments may help to understand the biology of these non-motor symptoms and test novel etiological hypothesis. At the current time, our understanding of these premotor symptoms is still in its infancy and the research calls for close multidisciplinary collaborations among clinicians, epidemiologists, basic scientists, and geneticists.

## References

[r1] Abbott RD, Petrovitch H, White LR, Masaki KH, Tanner CM, Curb JD (2001). Frequency of bowel movements and the future risk of Parkinson’s disease.. Neurology.

[r2] Abbott RD, Ross GW, Petrovitch H, Tanner CM, Davis DG, Masaki KH (2007). Bowel movement frequency in late-life and incidental Lewy bodies.. Mov Disord.

[r3] Abbott RD, Ross GW, White LR, Tanner CM, Masaki KH, Nelson JS (2005). Excessive daytime sleepiness and subsequent development of Parkinson disease.. Neurology.

[r4] Anderson G, Noorian AR, Taylor G, Anitha M, Bernhard D, Srinivasan S (2007). Loss of enteric dopaminergic neurons and associated changes in colon motility in an MPTP mouse model of Parkinson’s disease.. Exp Neurol.

[r5] Bach JP, Ziegler U, Deuschl G, Dodel R, Doblhammer-Reiter G (2011). Projected numbers of people with movement disorders in the years 2030 and 2050.. Mov Disord.

[r6] Berg D, Godau J, Seppi K, Behnke S, Liepelt-Scarfone I, Lerche S (2013). The PRIPS study: screening battery for subjects at risk for Parkinson’s disease.. Eur J Neurol.

[r7] Berg D, Marek K, Ross GW, Poewe W (2012). Defining at-risk populations for Parkinson’s disease: lessons from ongoing studies.. Mov Disord.

[r8] Bezard E, Yue Z, Kirik D, Spillantini MG (2012). Animal models of Parkinson’s disease: limits and relevance to neuroprotection studies.. Mov Disord.

[r9] Blesa J Phani S Jackson-Lewis V Przedborski S 2012 Classic and new animal models of Parkinson’s disease. J Biomed Biotechnol 2012:845618; 10.1155/2012/845618 PMC3321500 22536024

[r10] Boeve BF, Molano JR, Ferman TJ, Smith GE, Lin SC, Bieniek K (2011). Validation of the Mayo Sleep Questionnaire to screen for REM sleep behavior disorder in an aging and dementia cohort.. Sleep Med.

[r11] Boeve BF, Saper CB (2006). REM sleep behavior disorder: a possible early marker for synucleinopathies.. Neurology.

[r12] Bower JH, Grossardt BR, Maraganore DM, Ahlskog JE, Colligan RC, Geda YE (2010). Anxious personality predicts an increased risk of Parkinson’s disease.. Mov Disord.

[r13] Braak H, Tredici KD, Rüb U, de Vos RAI, Jansen Steur ENH, Braak E (2003). Staging of brain pathology related to sporadic Parkinson’s disease.. Neurobiol Aging.

[r14] Brämerson A, Johansson L, Ek L, Nordin S, Bende M (2004). Prevalence of olfactory dysfunction: the Skövde population-based study.. Laryngoscope.

[r15] Burke RE, Dauer WT, Vonsattel JP (2008). A critical evaluation of the Braak staging scheme for Parkinson’s disease.. Ann Neurol.

[r16] Caudle WM, Richardson JR, Wang MZ, Taylor TN, Guillot TS, McCormack AL (2007). Reduced vesicular storage of dopamine causes progressive nigrostriatal neurodegeneration.. J Neurosci.

[r17] Chen H, Zhang SM, Schwarzschild MA, Hernán MA, Ascherio A (2006). Survival of Parkinson’s disease patients in a large prospective cohort of male health professionals.. Mov Disord.

[r18] Claassen DO, Josephs KA, Ahlskog JE, Silber MH, Tippmann-Peikert M, Boeve BF (2010). REM sleep behavior disorder preceding other aspects of synucleinopathies by up to half a century.. Neurology.

[r19] Coelho M, Ferreira JJ (2012). Late-stage Parkinson disease.. Nat Rev Neurol.

[r20] de Lau LM, Koudstaal PJ, Hofman A, Breteler MM (2006). Subjective complaints precede Parkinson disease: the Rotterdam study.. Arch Neurol.

[r21] Dickson DW, Uchikado H, Fujishiro H, Tsuboi Y (2010). Evidence in favor of Braak staging of Parkinson’s disease.. Mov Disord.

[r22] Doty RL (2008). The olfactory vector hypothesis of neurodegenerative disease: Is it viable?. Ann Neurol.

[r23] Doty RL (2009). The olfactory system and its disorders.. Semin Neurol.

[r24] Doty RL, Shaman P, Kimmelman CP, Dann MS (1984). University of Pennsylvania Smell Identification Test: a rapid quantitative olfactory function test for the clinic.. Laryngoscope.

[r25] Drolet RE, Cannon JR, Montero L, Greenamyre JT (2009). Chronic rotenone exposure reproduces Parkinson’s disease gastrointestinal neuropathology.. Neurobiol Dis.

[r26] Dunning CJ, Reyes JF, Steiner JA, Brundin P (2012). Can Parkinson’s disease pathology be propagated from one neuron to another?. Prog Neurobiol.

[r27] Fang F, Xu Q, Park Y, Huang X, Hollenbeck A, Blair A (2010). Depression and the subsequent risk of Parkinson’s disease in the NIH-AARP Diet and Health Study.. Mov Disord.

[r28] Fearnley JM, Lees AJ (1991). Ageing and Parkinson’s disease: substantia nigra regional selectivity.. Brain.

[r29] Fernandez HH (2012). Nonmotor complications of Parkinson disease.. Cleve Clin J Med.

[r30] Fleming SM, Salcedo J, Fernagut PO, Rockenstein E, Masliah E, Levine MS (2004). Early and progressive sensorimotor anomalies in mice overexpressing wild-type human α-synuclein.. J Neurosci.

[r31] Fleming SM, Tetreault NA, Mulligan CK, Hutson CB, Masliah E, Chesselet MF (2008). Olfactory deficits in mice overexpressing human wildtype α-synuclein.. Eur J Neurosci.

[r32] Gao J, Huang X, Park Y, Hollenbeck A, Blair A, Schatzkin A (2011). Daytime napping, nighttime sleeping, and Parkinson disease.. Am J Epidemiol.

[r33] Gao X, Chen H, Schwarzschild MA, Ascherio A (2011). A prospective study of bowel movement frequency and risk of Parkinson’s disease.. Am J Epidemiol.

[r34] García-García F, Ponce S, Brown R, Cussen V, Krueger JM (2005). Sleep disturbances in the rotenone animal model of Parkinson disease.. Brain Res.

[r35] Goldstein DS (2010). Neuroscience and heart-brain medicine: the year in review.. Cleve Clin J Med.

[r36] Grenham S Clarke G Cryan JF Dinan TG 2011 Brain–gut–microbe communication in health and disease. Front Physiol 2 94; 10.3389/fphys.2011.00094 22162969 PMC3232439

[r37] Hawkes CH, Del Tredici K, Braak H (2007). Parkinson’s disease: a dual-hit hypothesis.. Neuropathol Appl Neurobiol.

[r38] Hawkes CH, Del Tredici K, Braak H (2009). Parkinson’s disease: the dual hit theory revisited.. Ann NY Acad Sci.

[r39] Hawkes CH, Del Tredici K, Braak H (2010). A timeline for Parkinson’s disease.. Parkinsonism Relat Disord.

[r40] Hummel T, Konnerth CG, Rosenheim K, Kobal G (2001). Screening of olfactory function with a four-minute odor identification test: reliability, normative data, and investigations in patients with olfactory loss.. Ann Otol Rhinol Laryngol.

[r41] Iranzo A, Molinuevo JL, Santamaría J, Serradell M, Martí MJ, Valldeoriola F (2006). Rapid-eye-movement sleep behaviour disorder as an early marker for a neurodegenerative disorder: a descriptive study.. Lancet Neurol.

[r42] Ishihara-Paul L, Wainwright NW, Khaw KT, Luben RN, Welch AA, Day NE (2008). Prospective association between emotional health and clinical evidence of Parkinson’s disease.. Eur J Neurol.

[r43] Jang H, Boltz D, McClaren J, Pani AK, Smeyne M, Korff A (2012). Inflammatory effects of highly pathogenic H5N1 influenza virus infection in the CNS of mice.. J Neurosci.

[r44] Jang H, Boltz D, Sturm-Ramirez K, Shepherd KR, Jiao Y, Webster R (2009). Highly pathogenic H5N1 influenza virus can enter the central nervous system and induce neuroinflammation and neurodegeneration.. Proc Natl Acad Sci USA.

[r45] Jellinger KA (2011). Synuclein deposition and non-motor symptoms in Parkinson disease.. J Neurol Sci.

[r46] Kudo T, Loh DH, Truong D, Wu Y, Colwell CS (2011). Circadian dysfunction in a mouse model of Parkinson’s disease.. Exp Neurol.

[r47] Kuo YM, Li Z, Jiao Y, Gaborit N, Pani AK, Orrison BM (2010). Extensive enteric nervous system abnormalities in mice transgenic for artificial chromosomes containing Parkinson disease-associated α-synuclein gene mutations precede central nervous system changes.. Hum Mol Genet.

[r48] Laloux C, Derambure P, Kreisler A, Houdayer E, Brueziere S, Bordet R (2008). MPTP-treated mice: long-lasting loss of nigral TH-ir neurons but not paradoxical sleep alterations.. Exp Brain Res.

[r49] Lam HA, Wu N, Cely I, Kelly RL, Hean S, Richter F (2011). Elevated tonic extracellular dopamine concentration and altered dopamine modulation of synaptic activity precede dopamine loss in the striatum of mice overexpressing human α-synuclein.. J Neurosci Res.

[r50] Lang AE (2011). A critical appraisal of the premotor symptoms of Parkinson’s disease: potential usefulness in early diagnosis and design of neuroprotective trials.. Mov Disord.

[r51] Leung FW (2007). Etiologic factors of chronic constipation: review of the scientific evidence.. Dig Dis Sci.

[r52] Leung L, Riutta T, Kotecha J, Rosser W (2011). Chronic constipation: an evidence-based review.. J Am Board Fam Med.

[r53] Li SX, Wing YK, Lam SP, Zhang J, Yu MW, Ho CK (2009). Validation of a new REM sleep behavior disorder questionnaire (RBDQ-HK).. Sleep Med.

[r54] Luk KC, Kehm V, Carroll J, Zhang B, O’Brien P, Trojanowski JQ (2012). Pathological α-synuclein transmission initiates Parkinson-like neurodegeneration in nontransgenic mice.. Science.

[r55] Marras C, Schule B, Munhoz RP, Rogaeva E, Langston JW, Kasten M (2011). Phenotype in parkinsonian and nonparkinsonian *LRRK2* G2019S mutation carriers.. Neurology.

[r56] McCrea GL, Miaskowski C, Stotts NA, Macera L, Varma MG (2009). A review of the literature on gender and age differences in the prevalence and characteristics of constipation in North America.. J Pain Symptom Manage.

[r57] McDowell K, Chesselet MF (2012). Animal models of the non-motor features of Parkinson’s disease.. Neurobiol Dis.

[r58] Mirelman A, Gurevich T, Giladi N, Bar-Shira A, Orr-Urtreger A, Hausdorff JM (2011). Gait alterations in healthy carriers of the LRRK2 G2019S mutation.. Ann Neurol.

[r59] Moberg PJ Kamath V Marchetto DM Calkins ME Doty RL Hahn CG 2013 Meta-analysis of olfactory function in schizophrenia, first-degree family members, and youths at-risk for psychosis. Schizophr Bull; 10.1093/schbul/sbt049 [Online 2 May 2013] PMC3885295 23641047

[r60] NINDS (National Institute of Neurological Disorders and Stroke). (2013). NINDS Common Data Elements: Parkinson’s Disease.. http://www.commondataelements.ninds.nih.gov/PD.aspx#tab=Data_Standards.

[r61] Noyce AJ, Bestwick JP, Silveira-Moriyama L, Hawkes CH, Giovannoni G, Lees AJ (2012). Meta-analysis of early nonmotor features and risk factors for Parkinson disease.. Ann Neurol.

[r62] Pan-Montojo F Anichtchik O Dening Y Knels L Pursche S Jung R 2010 Progression of Parkinson’s disease pathology is reproduced by intragastric administration of rotenone in mice. PLoS One 5 1 e8762; 10.1371/journal.pone.0008762 20098733 PMC2808242

[r63] Pan-Montojo FJ Funk RH 2010 Oral administration of rotenone using a gavage and image analysis of alpha-synuclein inclusions in the enteric nervous system. J Vis Exp 44 e2123; 10.3791/2123 PMC3280635 21085094

[r64] Pan-Montojo F Schwarz M Winkler C Arnhold M O’Sullivan GA Pal A 2012 Environmental toxins trigger PD-like progression via increased alpha-synuclein release from enteric neurons in mice. Sci Rep 2 898; 10.1038/srep00898 23205266 PMC3510466

[r65] Ponsen MM, Stoffers D, Twisk JW, Wolters E, Berendse HW (2009). Hyposmia and executive dysfunction as predictors of future Parkinson’s disease: a prospective study.. Mov Disord.

[r66] Postuma RB, Arnulf I, Hogl B, Iranzo A, Miyamoto T, Dauvilliers Y (2012a). A single-question screen for rapid eye movement sleep behavior disorder: a multicenter validation study.. Mov Disord.

[r67] Postuma RB, Gagnon JF, Vendette M, Desjardins C, Montplaisir JY (2011). Olfaction and color vision identify impending neurodegeneration in rapid eye movement sleep behavior disorder.. Ann Neurol.

[r68] Postuma RB, Gagnon JF, Vendette M, Fantini ML, Massicotte-Marquez J, Montplaisir J (2009). Quantifying the risk of neurodegenerative disease in idiopathic REM sleep behavior disorder.. Neurology.

[r69] Postuma RB, Lang AE, Gagnon JF, Pelletier A, Montplaisir JY (2012b). How does parkinsonism start? Prodromal parkinsonism motor changes in idiopathic REM sleep behaviour disorder.. Brain.

[r70] Postuma RB, Montplaisir JY, Pelletier A, Dauvilliers Y, Oertel W, Iranzo A (2012c). Environmental risk factors for REM sleep behavior disorder: a multicenter case-control study.. Neurology.

[r71] Reichmann H (2011). View point: Etiology in Parkinson’s disease. Dual hit or spreading intoxication.. J Neurol Sci.

[r72] Ross GW, Abbott RD, Petrovitch H, Tanner CM, Davis DG, Nelson J (2006). Association of olfactory dysfunction with incidental Lewy bodies.. Mov Disord.

[r73] Ross GW, Petrovitch H, Abbott RD, Tanner CM, Popper J, Masaki K (2008). Association of olfactory dysfunction with risk for future Parkinson’s disease.. Ann Neurol.

[r74] Savica R, Carlin JM, Grossardt BR, Bower JH, Ahlskog JE, Maraganore DM (2009). Medical records documentation of constipation preceding Parkinson disease: a case–control study.. Neurology.

[r75] Schenck CH Boeve BF Mahowald MW 2013 Delayed emergence of a parkinsonian disorder or dementia in 81% of older males initially diagnosed with idiopathic REM sleep behavior disorder (RBD): 16-year update on a previously reported series. Sleep Med 14 8 744 748; 10.1016/j.sleep.2012.10.009 23347909

[r76] Schenck CH, Bundlie SR, Mahowald MW (1996). Delayed emergence of a parkinsonian disorder in 38% of 29 older men initially diagnosed with idiopathic rapid eye movement sleep behaviour disorder.. Neurology.

[r77] Schintu N, Frau L, Ibba M, Caboni P, Garau A, Carboni E (2009). PPAR-gamma-mediated neuroprotection in a chronic mouse model of Parkinson’s disease.. Eur J Neurosci.

[r78] Schubert CR, Cruickshanks KJ, Fischer ME, Huang GH, Klein BE, Klein R (2012). Olfactory impairment in an adult population: the Beaver Dam Offspring Study.. Chem Senses.

[r79] Schubert CR, Cruickshanks KJ, Klein BE, Klein R, Nondahl DM (2011). Olfactory impairment in older adults: five-year incidence and risk factors.. Laryngoscope.

[r80] Shannon KM, Keshavarzian A, Dodiya HB, Jakate S, Kordower JH (2012). Is alpha-synuclein in the colon a biomarker for premotor Parkinson’s disease? Evidence from 3 cases.. Mov Disord.

[r81] Shiba M, Bower JH, Maraganore DM, McDonnell SK, Peterson BJ, Ahlskog JE (2000). Anxiety disorders and depressive disorders preceding Parkinson’s disease: a case-control study.. Mov Disord.

[r82] Shulman LM, Gruber-Baldini AL, Anderson KE, Vaughan CG, Reich SG, Fishman PS (2008). The evolution of disability in Parkinson disease.. Mov Disord.

[r83] Siderowf A, Jennings D, Connolly J, Doty RL, Marek K, Stern MB (2007). Risk factors for Parkinson’s disease and impaired olfaction in relatives of patients with Parkinson’s disease.. Mov Disord.

[r84] Siderowf A, Jennings D, Eberly S, Oakes D, Hawkins KA, Ascherio A (2012). Impaired olfaction and other prodromal features in the Parkinson At-Risk Syndrome Study.. Mov Disord.

[r85] Smith GA, Isacson O, Dunnett SB (2012). The search for genetic mouse models of prodromal Parkinson’s disease.. Exp Neurol.

[r86] Stiasny-Kolster K, Mayer G, Schafer S, Moller JC, Heinzel-Gutenbrunner M, Oertel WH (2007). The REM sleep behavior disorder screening questionnaire–a new diagnostic instrument.. Mov Disord.

[r87] Storch A, Schneider CB, Wolz M, Sturwald Y, Nebe A, Odin P (2013). Nonmotor fluctuations in Parkinson disease: Severity and correlation with motor complications.. Neurology.

[r88] Suares NC, Ford AC (2011). Systematic review: the effects of fibre in the management of chronic idiopathic constipation.. Aliment Pharmacol Ther.

[r89] Taylor TN, Caudle WM, Shepherd KR, Noorian A, Jackson CR, Iuvone PM (2009). Nonmotor symptoms of Parkinson’s disease revealed in an animal model with reduced monoamine storage capacity.. J Neurosci.

[r90] Tolosa E, Gaig C, Santamaria J, Compta Y (2009). Diagnosis and the premotor phase of Parkinson disease.. Neurology.

[r91] van Rooden SM, Colas F, Martínez-Martin P, Visser M, Verbaan D, Marinus J (2011). Clinical subtypes of Parkinson’s disease.. Mov Disord.

[r92] van Rooden SM, Visser M, Verbaan D, Marinus J, van Hilten JJ (2009). Motor patterns in Parkinson’s disease: a data-driven approach.. Mov Disord.

[r93] Vennemann MM, Hummel T, Berger K (2008). The association between smoking and smell and taste impairment in the general population.. J Neurol.

[r94] Wang L, Fleming SM, Chesselet MF, Tache Y (2008). Abnormal colonic motility in mice overexpressing human wild-type α-synuclein.. Neuroreport.

[r95] Wang L, Magen I, Yuan PQ, Subramaniam SR, Richter F, Chesselet MF (2012). Mice overexpressing wild-type human alpha-synuclein display alterations in colonic myenteric ganglia and defecation.. Neurogastroenterol Motil.

[r96] Weisskopf MG, Chen H, Schwarzschild MA, Kawachi I, Ascherio A (2003). Prospective study of phobic anxiety and risk of Parkinson’s disease.. Mov Disord.

[r97] Willis AW, Schootman M, Kung N, Evanoff BA, Perlmutter JS, Racette BA (2012). Predictors of survival in patients with Parkinson disease.. Arch Neurol.

[r98] Wilson RS, Arnold SE, Schneider JA, Boyle PA, Buchman AS, Bennett DA (2009). Olfactory impairment in presymptomatic Alzheimer’s disease.. Ann NY Acad Sci.

[r99] Wilson RS, Schneider JA, Arnold SE, Tang Y, Boyle PA, Bennett DA (2007). Olfactory identification and incidence of mild cognitive impairment in older age.. Arch Gen Psychiatry.

